# Whole-Exome Sequencing of Rare Site Endometriosis-Associated Cancer

**DOI:** 10.3390/diseases9010014

**Published:** 2021-02-04

**Authors:** Sonomi Kurose, Kentaro Nakayama, Sultana Razia, Masako Ishikawa, Tomoka Ishibashi, Hitomi Yamashita, Seiya Sato, Asuka Sakiyama, Shinya Yoshioka, Misa Kobayashi, Satoru Nakayama, Yoshiro Otuski, Noriyoshi Ishikawa, Satoru Kyo

**Affiliations:** 1 Department of Obstetrics and Gynecology, Shimane University School of Medicine, Izumo 693-8501, Japan; dokinchan.lj.1108@gmail.com (S.K.); raeedahmed@yahoo.com (S.R.); m-ishi@med.shimane-u.ac.jp (M.I.); tomoka@med.shimane-u.ac.jp (T.I.); memedasudasu1103@gmail.com (H.Y.); sato_seiya9534@yahoo.co.jp (S.S.); satoruky@med.shimane-u.ac.jp (S.K.); 2 Department of Obstetrics and Gynecology, Kobe City Medical Center Hospital, Kobe 650-0047, Japan; wishkitty.happy.914@gmail.com (A.S.); s.yoshioka@kcho.jp (S.Y.); 3 Department of Obstetrics and Gynecology, Seirei Hamamatsu Hospital, Hamamatsu 430-8558, Japan; misan_bobo@yahoo.co.jp (M.K.); satoru@sis.seirei.or.jp (S.N.); 4 Department of Organ Pathology, Seirei Hamamatsu Hospital, Hamamatsu 430-8558, Japan; kanatomo@med.shimane-u.ac.jp; 5 Department of Organ Pathology, Shimane University School of Medicine, Izumo 693-8501, Japan; otsuki@sis.seirei.or.jp

**Keywords:** rare site endometriosis-associated cancer, exome sequencing, actionable variants, immune checkpoint inhibitor

## Abstract

Malignant transformation of extraovarian endometriosis is rare, with the carcinogenesis mechanism unclear. To clarify the actionable variants of rare-site endometriosis-associated cancer (RSEAC), we performed whole-exome sequencing for the tumor, in two patients. The intestine was affected in both cases, although the histology was that of clear cell carcinoma and undifferentiated carcinoma, respectively. Therefore, the cases were referred to as endometriosis-associated intestinal tumors (EIATs). Actionable variants (all frameshift mutations) were identified in tumor suppressor genes *ARID1A*, *PTEN*, and *p53*; however, no oncogenic variants were identified. Both cases were microsatellite stable. The patient with undifferentiated carcinoma exhibited hypermutator and homologous recombination deficiency phenotypes. The dominant mutation signatures were signature 30 (small subset of breast cancers) and 19 (pilocytic astrocytoma) in patient 1, and signature 5 (small subset of breast cancers) and 3 (breast, ovarian, and pancreatic cancers) in patient 2. Immunohistochemistry revealed positive CD8 and PD-1 expression in both patients; patient 1 also showed positive PDL-1 expression. Our results suggest that RSEAC is associated with variants of tumor suppressor genes as epigenetic alterations. Mutation signature-based whole-exome sequencing could be useful to select an adjuvant chemotherapy regimen. High CD8 and PD-1 expression in RSEAC suggests that immune checkpoint inhibitors are useful for treatment.

## 1. Introduction

Endometriosis is a relatively common disease that occurs in the endometrial glands and stromal tissue outside the uterus. The incidence of endometriosis in women of reproductive age is estimated at 5–15% [1], and it most commonly affects the ovaries, fallopian tubes, cul-de-sac, and uterine cervix. Uncommon sites (i.e., extragonadal sites) include the intestine, urinary tract, lungs, skin, and central nervous system [2]. 

Although endometriosis is invasive and metastasis can occur, the condition is determined as benign. However, recent etiological and molecular evidence suggests that endometriosis can progress in a step-wise manner from benign to borderline to malignant [3]. The incidence of the malignant transformation of endometriosis reaches up to 1% in Japanese women [4]. These tumors usually arise from the ovaries; however, 21.3% seem to occur in extragonadal sites, and endometriosis-associated intestinal tumors (EAITs) are even rarer [5].

Molecular analyses of endometriosis-associated ovarian cancers are frequently reported in the literature [6,7,8,9], but there has been no molecular investigation of rare site endometriosis-associated cancer (RSEAC) to date. Because the microenvironment of the ovary differs from those of other organs with regard to the effects of hormones, the carcinogenic drivers may be different between the malignant transformation of ovarian endometriosis and extragonadal endometriosis. In particular, in ovarian endometriosis, direct hormonal effects can occur even after menopause [10]. Therefore, to clarify the molecular driver events in the malignant transformation of endometriosis of extragonadal sites, whole-exome sequencing was performed for two cases of EAITs. These findings can provide new insights into the underlying molecular driver events specific to RSEAC, which can be used to determine specific treatment options.

## 2. Materials and Methods

### 2.1. Tumor Samples and DNA Extraction

**Figure 1:**
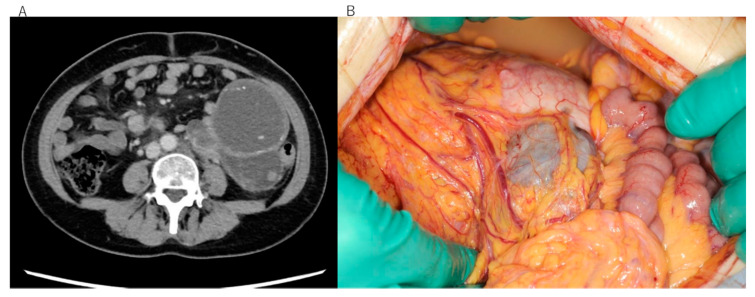

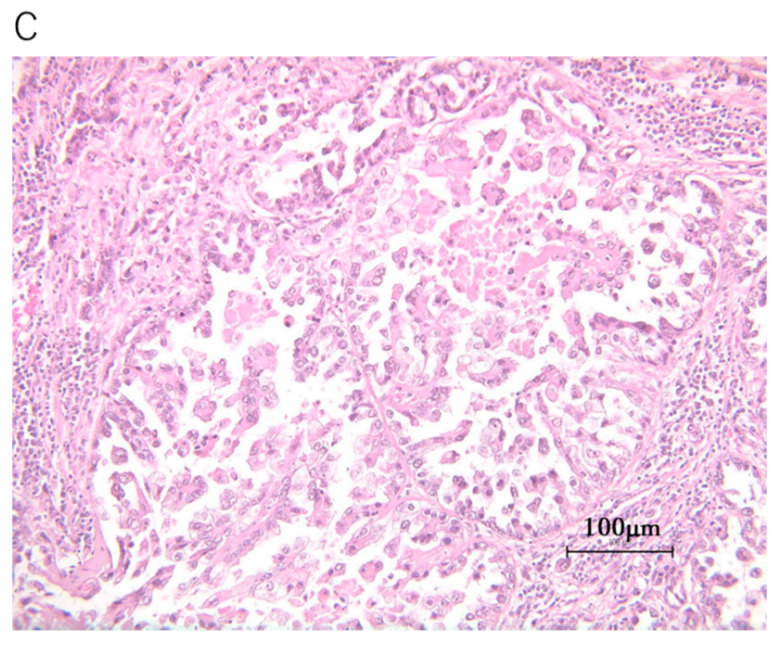
Clinical and pathological images of the rare-site endometriosis-associated cancer (RSEAC) in patient 1. (**A**) Computed tomography scan showing a giant abdominal tumor. (**B**) Surgical findings showing RSEAC arising from the intestine. (**C**) Hematoxylin and eosin staining showing clear cell carcinoma.

**Figure 2:**
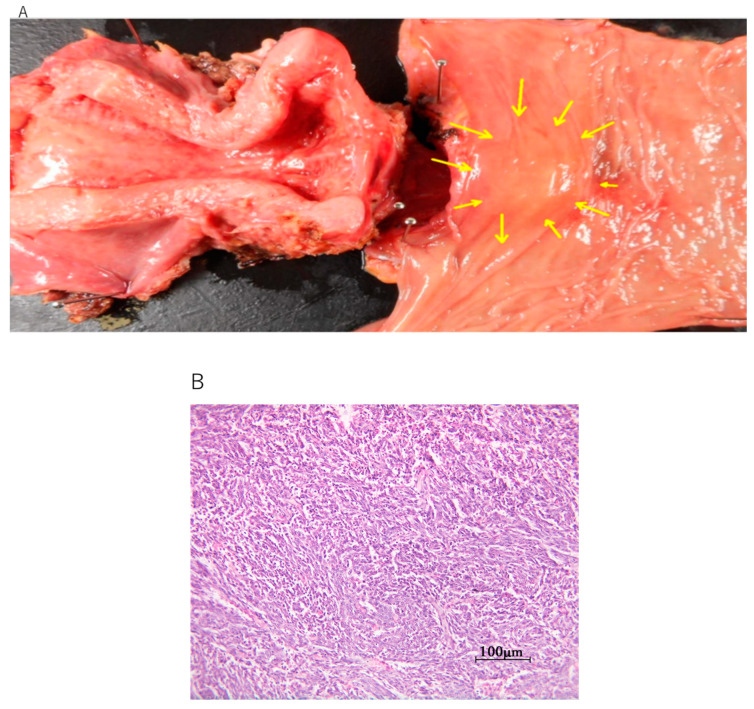
Clinical and pathological images of the rare-site endometriosis-associated cancer (RSEAC) in patient 2. (**A**) Surgically dissected uterus and rectum; yellow arrows indicate the location of the RSEAC. (**B**) Hematoxylin and eosin staining showing undifferentiated carcinoma.

This study included two patients with EIATs, one from Seirei Hamamatsu General Hospital and one from Kobe City Medical Center Hospital. The diagnosis was malignant transformation of extragonadal endometriosis in both cases, according to criteria recommended by a Japanese survey [11]. Histologically, the tumor in patient 1 was identified as clear cell carcinoma arising in the left sigmoid colon (Figure 1A–C), whereas that of patient 2 was identified as undifferentiated carcinoma arising in the rectum (Figure 2A,B). Neither patient received hormone therapy before these cancers were diagnosed (Table 1). The patients’ characteristics are summarized in Table 1. Clinical information was obtained retrospectively from electronic medical records. The study was conducted in accordance with the tenets of the Declaration of Helsinki and Title 45 (United States Code of Federal Regulations), Part 46 (Protection of Human Subjects), effective 13 December 2001.

Hematoxylin and eosin-stained tissue sections were reviewed and marked with lines to separate the carcinoma portion and stromal portion by a skilled gynecological pathologist (N.I.). The sections were placed on membrane slides and counterstained with hematoxylin. Selected tumor tissues (10-mm-thick sections) were manually dissected to obtain a high percentage of tumor cells. After 48 h of digestion with a proteinase, DNA was extracted from the macrodissected samples using QIAmp DNA Micro Kit (Qiagen, Valencia, CA, USA) according to the manufacturer’s instructions. We confirmed that the carcinoma/stroma ratio was more than 70% for each sample. A portion of the normal intestine from each patient was used as a reference control.

### 2.2. Exome Sequencing

Next-generation sequencing of the EIATs was performed as previously reported [12]. In brief, DNA integrity was first checked by calculating the DNA unity number using Agilent 2000TapeStation (Agilent Technologies, Santa Clara, CA, USA) prior to targeted amplicon whole-exome sequencing with the Illumina MiSeq sequencing platform (Illumina, San Diego, CA, USA). The sequencing files were analyzed in the GenomeJack bioinformatics pipeline (Mitsubishi Space Software, Tokyo, Japan). Cancer-specific alterations in somatic genes, including single-nucleotide variations, insertions/deletions, and gene copy number alterations, were obtained. The tumor mutational burdens were also determined.

### 2.3. Immunohistochemical Analysis

The protein expression levels of CD8, PDL-1, and PD-1 were judged by immunohistochemical analysis using antibodies against CD-8 (1:100; SP57, Roche, Basel, Switzerland), PD-L1 (ab205921, Abcam, Cambridge, United Kingdom), and PD-1 (SP263, Roche). The methods of immunohistochemistry and criteria of positive staining of each protein have been previously described [13]. Two gynecologic oncologists (S.K. and K.N.), who were blinded to the clinicopathologic parameters, evaluated the samples under a microscope.

## 3. Results

**Figure 3:**
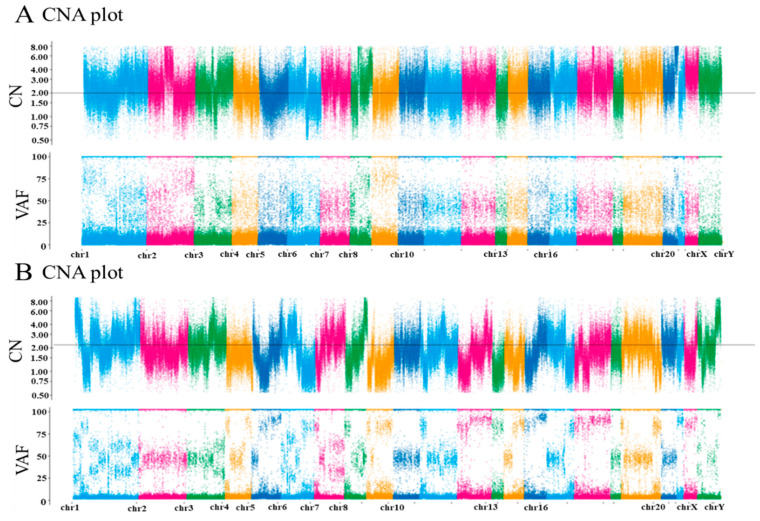
Copy number (CN) plots of rare-site endometriosis-associated cancer (RSEACs). The horizontal axis shows the chromosome location, and the vertical axis shows the gene CN. (**A**) Representative CN plot of patient 1, which appears normal. (**B**) Representative CN plot of patient 2, showing a high frequency of gene amplification and deletions, indicating homologous recombination deficiency (HRD). VAF, variant allele frequency.

Whole-exome sequencing was performed on genomic DNA from EIAT tissues, which was compared with the results from normal intestine tissues. In patient 1, *ARID1A* and *PTEN* frameshift mutations were identified as actionable variants. In patient 2, a *p53* frameshift mutation was identified as an actionable variant. Interestingly, these three genes are tumor suppressor genes, and no actionable variants were identified in known oncogenes. Variant position and variant allele frequencies are summarized in Table 2. The other candidate actionable variants identified in these cases are summarized in Supplementary Table S1 and Table S2. These variants were considered to have uncertain significance rather than being pathogenic. No copy number alterations in oncogenes or tumor suppressor genes were identified in patient 1. In contrast, patient 2 showed many copy number alterations, including *MYC* amplification and loss of heterozygosity (LOH) of *ARID1A*, *BRCA2*, and *RAD51* (Supplementary Table S3). The copy number plot of patient 1 was normal. However, the copy number plot of patient 2 indicated homologous recombination deficiency (HRD), and the existence of numerous LOH and amplification genes (Figure 3, Supplementary Table S3). Both patients were microsatellite stable, and 138/49,722,613 and 193/49,845,875 non-synonymous single-nucleotide variants (SNVs) were identified in patient 1 and patient 2, respectively. Identification of more than 200 non-synonymous SNVs indicates the possibility of a hypermutator phenotype. Patient 2 showed a relatively high frequency of non-synonymous SNVs, suggesting that this tumor might have a hypermutator phenotype.

**Figure 4:**
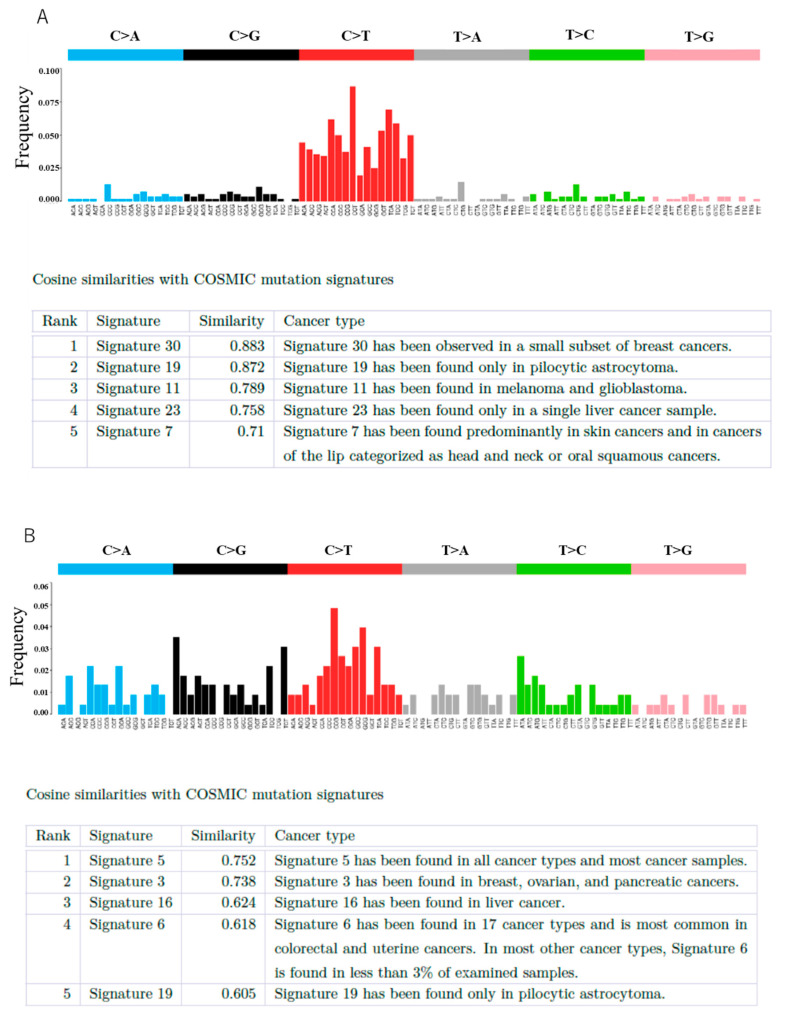
Cosine similarities with COSMIC mutation signatures. (**A**) Signature 30 showed the highest similarities with the mutation profile of patient 1. (**B**) Signature 5 showed the highest similarities with the mutation profile of patient 2. Similarity index was defined as 0 dissimilar, 1 identical.

**Figure 5:**
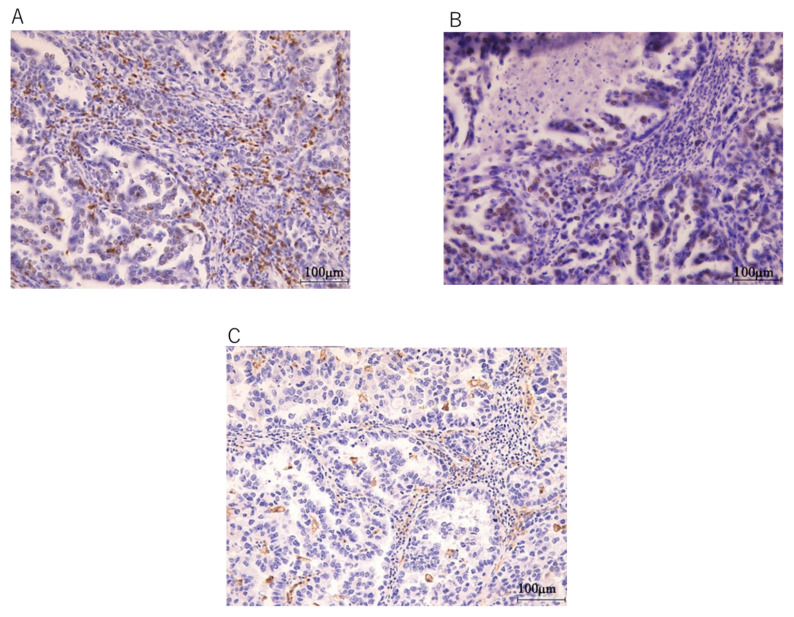
Immunohistochemical analysis of CD8, PD-1, and PDL-1 in RSEACs. (**A**) CD8 expression score 2+. (**B**) PD1 expression score 2+. (**C**) PDL-1 expression score 1+.

Next, we aimed to determine the types of mutation signatures of the EIATs. The dominant mutation signatures of patient 1 were signature 30 (a small subset of breast cancers) and 19 (pilocytic astrocytoma), whereas those for patient 2 were signature 5 (a small subset of breast cancers) and 3 (breast, ovarian, and pancreatic cancers) (Figure 4). Immunohistochemical analysis revealed that both patients were positive for CD8 and PD-1 expression, and only patient 1 was positive for PDL-1 expression. Immunohistochemical findings of case 1 are shown in Figure 5.

## 4. Discussion

Malignant transformation of extragonadal endometriosis is extremely rare, and several case reports, series, and reviews have focused on the disease [14,15,16,17,18,19,20]. However, the incidence and treatment are still poorly understood. Very recently, a Japanese nationwide survey analyzed the etiology and incidence of this rare condition, which resulted in the name “RSEAC” and estimated its incidence to be 0.79% (11 RSEACs identified among 1397 extragonadal endometriosis cases at less common/rare sites over 10 years) [11]. Although prolonged unopposed estrogen therapy is considered a risk factor for RSEAC [14,21], the two patients in this study had never received hormone therapy. Several researchers have reported the frequency of endometriosis-related ovarian neoplasms (ERONs) in women to be 0.72–17.0% [4,22,23]. Although carcinogenic driver events of endometriosis-associated ovarian cancers are well understood [6,7,8,9], molecular genetic analysis of RSEACs has not been performed until now. Moreover, the driver genes of the carcinogenesis of RSEACs have not been identified. Actionable variants of oncogenes, such as *KRAS* and *PIK3CA*, are frequently determined in both ERONs and colorectal cancers [6,24]. However, we identified only pathogenic variants of tumor suppressor genes, such as *ARID1A*, *PTEN*, and *p53*, by whole-exome sequencing, and no actionable variants of oncogenes were determined in our analysis. These differences may be due to the organ-specific carcinogenic event, especially in ERONs; ovarian endometriosis was directly affected by hormones even after menopause [10]. Furthermore, we could not find druggable variants in this study.

Using a mathematical approach with next-generation sequencing data, Vogelstein et al. [25,26] reported that at least three mutations were necessary to cause human colon and lung cancers. Our current findings from whole-exome sequencing data of two RSEACs conflict with this result. Taken together, the current findings and previous reports suggest that epigenetic alterations may be important carcinogenic events in RSEACs.

Recently, recurrent mutations affecting *KRAS* and *PIK3CA* have been reported in ovarian endometriosis, deep infiltrative endometriosis, uterine adenomyosis, normal endometrium, and iatrogenic endometriosis (i.e., extragonadal endometriosis), suggesting that driver mutations of oncogenes are likely to exist in RSEACs [27,28,29,30,31,32,33,34,35]. However, we were unable to detect any driver oncogene mutations in RSEACs, which may be related to its very low incidence, with only 11 cases having been identified during the past 10 years in Japan [11].

Surprisingly, whole-exome sequencing revealed that patient 2 showed both the potential hypermutator phenotype and HRD phenotype. Since these two phenotypes are usually mutually exclusive, this finding suggests that a subset of RSEAC may have a specific genetic background. The microenvironments of ERONs and RSEACs are different, especially because ERONs are exposed to ovarian hormones directly even after menopause [10]. Therefore, we hypothesized that the carcinogenic driver events differ between them; however, no strict conclusion can be obtained at this point owing to the rarity of RSEACs.

There is typically no standardized therapy for rare malignant tumors because of their rarity and consequent lack of clinical trials. Several case reports have emphasized the efficacy of platinum and taxane combination chemotherapy against RSEACs [36,37]. Very recently, cancer panel sequencing for rare malignant tumors such as adenoid cystic carcinoma of Bartholin’s glands and castration-resistant prostate cancer was reported [38,39]. However, there are no reports of RSEAC clinical sequences, and it is unclear which cancer is genetically similar. Therefore, the mutation signatures obtained for these two cases from whole-exome sequencing may be useful for selecting an adjuvant chemotherapy regimen according to the type of cancer that the mutation signature indicates. A large multi-institutional prospective study with mutation signature analysis is required to standardize the chemotherapy regimen for RSEACs.

Recently, targeting the immune escape mechanisms of cancer cells and immune checkpoint inhibitor treatment have become the standard for cancer therapy [40]. Both a high frequency of tumor-infiltrative T cells and PD-1 expression are thought to be potential biomarkers for indicating a response to immune checkpoint inhibitors [41,42]. The high frequency of tumor-infiltrating T cells (revealed by immunostaining of CD8) and the high frequency of PD-1 expression in tumor-infiltrating lymphocytes in the current cases of RSEAC suggest that immune checkpoint inhibitor therapy could also be effective for patients with RSEAC. Patient 2 showed the potential hypermutator phenotype; therefore, abundant neoantigen might be expressed. However, patient 1 did not show any genetic evidence suggesting high tumor-infiltrating T cell expression. It is presumed that the immune system is more likely to detect RSEAC since the tumor is present at a site that should not have existed.

The current study has several limitations. First, only two cases could be analyzed because of the rarity of the disease. Therefore, a study with a higher number of samples is required to validate these findings. This will enable a statistical analysis of the relationship between the variants identified in the current study and patient therapy outcomes. Second, we identified only genetic mutations via next-generation sequencing; therefore, the types of carcinogenic events assessed were limited. Further analyses using gene microarray, microRNA array, and chromatin remodeling will also be needed to determine the complete molecular mechanism that underlies progression to RSEACs.

## 5. Conclusions

The current findings suggest that the actionable variants in RSEACs are mainly tumor suppressor genes such as *ARID1A*, *PTEN*, and *p53*. No actionable variants of oncogenes were identified in these two cases of RSEACs. Carcinogenesis of RSEAC might be based on variants of tumor suppressor genes along with epigenetic alterations. Mutation signatures based on whole-exome sequencing could be useful to select an adjuvant chemotherapy regimen. Furthermore, the two present RSEAC patients showed high frequencies of infiltrating T cell (CD8) expression and high PD-1 expression in infiltrating T cells in the tumor, suggesting that immune checkpoint inhibitors are useful for treating these types of cancers.

## Figures and Tables

**Table 1:** Clinicopathological information of rare-site endometriosis-associated cancer (RSEACs).

Case No.	Age	Parity	Hormone Therapy	Location	Surgical Operation	Chemotherapy	Histology	Survival Time	Prognosis
1	52	0	None	Descending colon	TAH+BSO+ Omentectomy +left hemicolectomy	TC 9course, PLD +Bev 3 course	Clear cell carcinoma	15 months	DOD
2	68	2	None	Rectum	TLH+BSO+LLAR	TC 3course	Undifferentiated carcinoma	6 months	AWD

**Table 2:** Actionable variants in rare-site endometriosis-associated cancer (RSEACs).

Case No.	Actionable Gene Variants	Variant Allele Frequency (%)	Copy Number
1	*ARID1A* M835Wfs*24	51.3	2
	*PTEN* D19Rfs*26	32.5	2.3
2	*p53* R259fs*6	76.6	1.9

## Data Availability

Data of current study was available from corresponding author (K.N.).

## Supplementary Materials

The following are available online at https://www.mdpi.com/2079-9721/9/1/14/s1, Table S1. Candidate actionable variants in case 1. Table S2. Candidate actionable variants in case 2. Table S3. Gene list of copy number alteration in case 2.

## Abbreviations

TAH	Total abdominal hysterectomy
BSO	Bilateral salping oohprectomy
TLH	Total laparoscopic hysterectomy
LLAR	Laparoscopic low anterior resection
TC	Taxan + carboplatin
PLD	Doxorubicin hydrochloride liposome
DOD	Died of disease
AWD	Alive without disease
VAF	Variant allele frequency
CN	Copy number
