# Supplementary material for: Whole-Exome Sequencing of Rare Site Endometriosis-Associated Cancer
Source: Diseases. 2021 Feb 4;9(1):14. doi: 10.3390/diseases9010014 (PMC7931088; doi:10.3390/diseases9010014)
Supplement: Supplementary file 1 [file diseases-09-00014-s001.pdf]

Supplementary

Whole-Exome Sequencing of Rare Site Endometriosis-Associated Cancer

Sonomi Kurose [1], Kentaro Nakayama [1,*], Sultana Razia [1], Masako Ishikawa [1], Tomoka Ishibashi [1], Hitomi Yamashita [1], Seiya Sato [1], Asuka Sakiyama [5], Shinya Yoshioka [5], Misa Kobayashi [3], Satoru Nakayama [3], Yoshiro Otuski [4], Noriyoshi Ishikawa [2] and Satoru Kyo [1]

Supplementary Table 1. Candidate actionable variants in case 1.

Function	Gene variants	VAF (%)	CN
OG	*CTNNB1* V358A	22.2	2.4
OG	*MNX1* L34F	24.8	2.4
OG	*TFG* G241D	25	1.7
OG	*ZCCHC8* A47P	36	2.5
OG	*MUC4* T3718A	47.2	3
TSG	*SMC1A* R771Q	35.7	2.8
TSG	*FBXO11* L560F	21.1	2.5

VAF: Variant allele frequency

CN: Copy number

OG: Oncogene

TSG: Tumor suppressor gene

Supplementary Table 2. Candidate actionable variants in case 2.

Function	Gene variants	VAF (%)	CN
OG	*USP* R763W	8	1.8
OG	*MUC16* P563T	40.7	2.1
OG	*USP8* N764K	8	1.8
TSG	*PRF1* C407Y	40.8	1.9

VAF: Aariant allele frequenct

CN: Copy number

OG: Oncogene

TSG: Tumor suppuressor gene

Supplementary Table 3. Gene list of copy number alteration in case 2

Function	Gene name	CNA	Copy number
dMMR	PMS2	LOH	1.2
HRD	*ARID1A*	LOH	1.1
HRD	*BRCA2*	LOH	1
HRD	*CHEK2*	LOH	1.1
HRD	*FANCA*	LOH	1.1
HRD	*FANCC*	LOH	1
HRD	*RAD50*	LOH	0.9
HRD	*RAD51*	LOH	1
HRD	*WRN*	LOH	1.2
OG	*CRLF2*	Amp	4.4
OG	*ELF4*	Amp	4.4
OG	*MTCP1*	Amp	4.8
OG	*MYC*	Amp	4.2
OG	*P2RY8*	Amp	4.4
OG	*PRDM16*	Amp	6
TSG	*BUB1B*	LOH	1
TSG	*CDKN1B*	LOH	0.8
TSG	*STK11*	LOH	1.2
TSG	*ERCC5*	LOH	1
TSG	*B2M*	LOH	1
TSG	*NCOR1*	LOH	1
TSG	*TSC1*	LOH	1
TSG	*RB1*	LOH	1
TSG	*RBM10*	LOH	1.1
TSG	*KDM6A*	LOH	1.1
TSG	*NF2*	LOH	1.1
TSG	*NF1*	LOH	1
TSG	*CNOT3*	LOH	1.1
TSG	*FLCN*	LOH	1
TSG	*CDKN2A*	LOH	1
TSG	*SMARCB1*	LOH	1.1
TSG	*PIK3R1*	LOH	0.9
TSG	*KMT2D*	LOH	0.8
TSG	*ARID2*	LOH	0.8
TSG	*XPA*	LOH	1
TSG	*CDH1*	LOH	1.1
TSG	*BCOR*	LOH	1.1
TSG	*EP300*	LOH	1.1
TSG	*SMARCD1*	LOH	0.8
TSG	*SDHB*	LOH	1.1

TSG	*ARID1B*	LOH	1
TSG	*TNFAIP3*	LOH	1
TSG	*APOBEC3B*	LOH	1.1
TSG	*PRDM1*	LOH	1

dMMR: Deficient mismatch repair

HRD: Homologous recombination deficiency

OG: Oncogene

TSG:Tumor suppressor gene
